# The Beta-adrenergic agonist, Ractopamine, increases skeletal muscle expression of Asparagine Synthetase as part of an integrated stress response gene program

**DOI:** 10.1038/s41598-018-34315-9

**Published:** 2018-10-29

**Authors:** David Brown, Kevin Ryan, Zoe Daniel, Molebeledi Mareko, Richard Talbot, Joanna Moreton, Tom C. B. Giles, Richard Emes, Charlie Hodgman, Tim Parr, John M. Brameld

**Affiliations:** 1 School of Biosciences, University of Nottingham, Sutton Bonington Campus, Loughborough, LE12 5RD UK; 2 School of Veterinary Medicine and Science, University of Nottingham, Sutton Bonington Campus, Loughborough, LE12 5RD UK; 3 Advanced Data Analysis Centre, University of Nottingham, Sutton Bonington Campus, Loughborough, LE12 5RD UK; 4 The Roslin Institute and Royal (Dick) School of Veterinary Studies, University of Edinburgh, Midlothian, EH25 9RG UK; 5 Present Address: RenaSci Limited, BioCity, Pennyfoot Street, Nottingham, NG1 1GF UK; 6 Present Address: Genetics, Guy’s Hospital, London, SE1 9RT UK; 7 Present Address: Botswana College of Agriculture, Gaborone, Botswana

**Keywords:** Target identification, Gene expression profiling, Animal physiology

## Abstract

Synthetic beta-adrenergic agonists (BA) have broad biomedical and agricultural application for increasing lean body mass, yet a poor understanding of the biology underpinning these agents is limiting further drug discovery potential. Growing female pigs (77 ± 7 kg) were administered the BA, Ractopamine (20 ppm in feed), or the recombinant growth hormone (GH), Reporcin (10 mg/48 hrs injected) for 1, 3, 7, 13 (*n* = 10 per treatment, per time point) or 27 days (*n* = 15 per treatment). Using RNA-sequencing and inferred pathway analysis, we examined temporal changes to the *Longissimus Dorsi* skeletal muscle transcriptome (*n* = 3 per treatment, per time point) relative to a feed-only control cohort. Gene expression changes were affirmed by quantitative-PCR on all samples (*n* = 164). RNA-sequencing analysis revealed that BA treatment had greater effects than GH, and that asparagine synthetase (*Asns*) was the 5^th^ most significantly increased gene by BA at day 3. ASNS protein expression was dramatically increased by BA treatment at day 7 (p < 0.05). The most significantly increased gene at day 3 was activating transcription factor 5 (*Atf5*), a transcription factor known to regulate ASNS gene expression. Gene and protein expression of *Atf4*, another known regulator of *Asns* expression, was not changed by BA treatment. Expression of more than 20 known *Atf4* target genes were increased by BA treatment, suggesting that BA treatment induces an integrated stress response (ISR) in skeletal muscle of pigs. In support of this, mRNA expression of sestrin-2 (*Sesn2*) and cyclin-dependant kinase 1 alpha (*Cdkn1a*), two key stress-responsive genes and negative regulators of cellular growth, were also strongly increased from day 3 of BA treatment. Finally, tRNA charging was the most significantly enriched pathway induced by BA treatment, suggesting alterations to the translational capacity/efficiency of the muscle. BA-mediated changes to the skeletal muscle transcriptome are highly indicative of an integrated stress response (ISR), particularly genes relating to amino acid biosynthesis and protein translational capacity.

## Introduction

Synthetic beta-adrenergic agonists (BA) have broad biomedical and agricultural application; from combating muscle wasting to accelerating the accretion of lean mass in livestock^1–3^. BA stimulate accretion of myofibrillar proteins in adult skeletal muscle by increasing protein synthesis and/or decreasing protein degradation^4–6^. Despite their broad application and well-documented efficacy, a poor understanding of the intricate biology underpinning the physiological actions of BA is limiting further drug discovery potential in this field.

BA mediate their effects on skeletal muscle via beta-adrenergic receptors (predominantly the beta-2 subtype)^1^, activating the adenylate cyclase pathway and inducing an increase in intracellular cyclic adenosine monophosphate (cAMP) concentrations. Elevated cAMP activates Protein Kinase A (PKA)-dependant phosphorylation of enzymes such as glycogen phosphorylase, which results in the rapid mobilization of muscle glycogen stores^3^. PKA also phosphorylates the cAMP-Responsive Element Binding Proteins (CREBs, also known as activating transcription factors (ATF))^7^. Given that CREB/ATF transcription factors occupy approximately 20% of human protein-coding gene promoters^8^, it is perhaps unsurprising that BA trigger extensive alterations to the skeletal muscle transcriptome^9,10^. The Protein Kinase B (AKT) – Mechanistic Target of Rapamycin (mTOR) pathway, a major regulator of protein synthesis and cellular metabolism^11^, has been implicated in the muscle sparing and muscle hypertrophy effects induced by BA^12^. Increased calpastatin expression and reduced calpain activity have also been reported in BA treated animals^4,5,13^, which may account for the observed reductions in skeletal muscle proteolysis^6^.

Identifying genes that are involved in the muscle modifying effects of BA could infer novel modes of muscle mass regulation, enabling the identification of new strategies to manipulate muscle mass. Two previous studies examined time-dependant responses of the skeletal muscle transcriptome to BA administration following 1 and 4 hours^10^ or 1 and 10 days^9^ of administration. These studies revealed impressive alterations in gene expression by BA, but the strength of the data set was limited by the use of only 2 time points. We recently^14^ expanded on these studies, employing a pig microarray to analyse temporal changes to the pig skeletal muscle transcriptome in response to BA and growth hormone (GH) administration spanning ~4 weeks, using time points at 1, 3, 7, 13 and 27 days, which, to date, is the most comprehensive data set of its kind. In that study^14^, we identified coordinate increases in expression of mitochondrial phosphoenolpyruvate carboxykinase 2 (PEPCK-M) and 3-phosphoglycerate dehydrogenase (PHGDH) in response to BA. These enzymes are both involved in amino acid biosynthesis and their function in adult skeletal muscle is currently being explored. We attributed the identification of these enzymes to the use of such an extensive time course; however, the microarray employed did not provide full coverage of the pig transcriptome. Therefore, in line with NC3R guidelines to reduce the number of animals used in research^15^, we performed RNA-sequencing on the same samples^14^ to obtain better coverage of the skeletal muscle transcriptome in response to GH or BA treatment. The aim being to investigate global alterations in skeletal muscle gene expression during administration of GH or the synthetic BA, Ractopamine, and thereby identify novel molecular events underpinning the “muscle modifying” effects of this anabolic compound. Given the increasing use of the pig as a biomedical model for human research^16^, this study provides biologically relevant information to both biomedical and agricultural fields.

## Methods

### Pig study design

For a full description of the study design, see^14^ from which the samples used herein were generated. The project was approved by The University of Nottingham Ethical Review Committee and conducted in accordance with the UK Animals (Scientific Procedures) Act of 1986 (Project License PPL 40/3010). Briefly, adult gilts (female pigs) with a mean starting body weight of 77 ± 7 kg were all provided a high quality feed (non-pelleted, 16.7% protein finisher feed, 14 MJ/kg; Target Feeds, UK). The BA group received the beta-adrenergic agonist, Ractopamine (Elanco Animal Health, Grenfield, IN), at 20 ppm in their feed, whereas the GH group were administered an intramuscular injection of the recombinant growth hormone, Reporcin (Zamira; 10 mg/kg/48-hours, GH group) for 1, 3, 7, 13 (*n* = 10 per treatment per time point) or 27 days (*n* = 15 per treatment). Treated animals were compared to a control cohort (receiving feed only) at each respective time point. After an overnight fast, animals were euthanized by electrical stunning followed by exsanguination. A sample of the *Longissimus Dorsi* (LD) muscle was excised at the 10^th^ rib and RNA extracted as previously described^14^. LD muscle RNA from 3 randomly selected pigs per treatment per time point (*n*  = 45 pigs) was used for quantitative RNA-sequence analyses. Validation of RNA-sequencing data was performed by quantitative-PCR (qPCR) on all 164 samples.

### Preparation of RNA

Total RNA was extracted from 100 mg of frozen LD muscle tissue from all 164 pigs using TRIZOL reagent (Invitrogen, Paisley, UK) followed by DNase treatment (Promega, Southampton, UK), according to the manufacturer’s instructions. The RNA samples selected for RNA-sequencing were purified using the Qiagen miRNeasy kit, by following the manufacturer’s instructions. RNA quantity was measured using a Nanodrop ND-1000 (Nanodrop Technologies, Wilmington, US) and RNA integrity number (RIN) was determined using an Agilent 2100 Bioanalyser (Agilent, Stockport, UK). Samples used for RNA-sequencing had a minimum RIN of 7, with an average RIN of 8.2.

### RNA-sequencing

RNA sequencing was carried out using the Illumina platform. Sequencing libraries for each sample were prepared using the TruSeq RNA Sample Prep Kit-v2 from Illumina (Cambridge UK). Briefly, poly(A) RNA was captured from an input of 1 µg of total RNA using the beads provided in the kit, the chemical fragmentation of the RNA is included in the elution of the poly(A) RNA from the beads. The fragmented RNA was reverse transcribed using a random primer cocktail provided in the kit and SuperScript II (Life Technologies, Paisley UK). A double stranded DNA generated from the cDNA was blunt ended before A-tailing and the ligation of barcoded adapters. After removing un-ligated adapters the library was subjected to 11 cycles of PCR to increase the yield of products.

The final sequencing libraries were assessed using electrophoresis on an Agilent Bioanalyser DNA 1000 chip (Agilent, Stockport, UK) to identify the size of the library products; the average library size was 328 ± 8 bases. The sequencing library was quantified by qPCR using the KAPA Library Quantification Kits for Illumina platforms (Kapa Biosystems, London, UK); the average concentration of the libraries was 14.2 nM ± 7.7 nM. Balanced library pools of 9 samples per pool were prepared and quantified by qPCR.

Sequencing of the libraries was carried out on an Illumina HiSeq 2500 in Rapid output mode using the Illumina Truseq Rapid v1 chemistry. The library pools were denatured and loaded at a concentration of 13 pM onto the flow cell using the Illumina cBot with the Truseq Rapid PE clustering kit. Libraries were sequenced for 100 bases paired-end sequencing providing an average of 25 million reads per sample for analysis. Data is available at the European Nucleotide Archive (https://www.ebi.ac.uk/ena/) Project PRJEB28262.

### Differential gene expression and inferred pathway enrichment analysis

Fastq format reads were processed using Trim Galore! to remove adapter sequences and low quality bases (Phred < 20). Trimmed reads were mapped to the pig genome Ensembl build (Sscrofa10.2.73, Aug 2011 Ensembl v73) using TopHat v2.0.9^17^ using the option ‘--no-novel-juncs’ to quantify the reference annotation only. Mapped read counts for each annotated gene were generated using htseq-count (version 0.5.4p3, options --stranded = no –a10)^18^. Significantly differentially expressed genes between control and treated at each time point were identified using EdgeR^19^, which uses empirical Bayes estimation and exact tests based on the negative binomial distribution to identify statistically robust differentially expressed genes.

False discovery rate (FDR) was calculated using the Benjamini-Hochberg correction^20^. Identified differentially expressed genes (FDR corrected p-value < 0.05) were assessed for enrichment of pathways and annotations within these genes. Statistical enrichment was calculated by a right tailed Fisher’s exact test (IPA, QIAGEN Redwood City www.qiagen.com/ingenuity). Results were visualised by plotting the enrichment in different groups as –log10 p values.

### Quantitative PCR validation of mRNA abundance

Transcript abundance of differentially expressed genes (identified by RNA-sequencing analysis) was validated by qPCR on all 164 LD muscle samples (control, BA and GH treated) using the standard curve method and expression values were normalized to total cDNA in the PCR reaction using the established oligreen method as previously described^14^. The inclusion of GH-treated samples in the gene expression validation permitted comparison of effects between BA and GH. Sample treatment and time allocations were blinded to the researcher conducting the qPCR. All oligonucleotide primer sequences are displayed in Table 1.

**Table 1:** Oligonucleotide sequences used for quantitative PCR.

Gene ID	Forward	Reverse
*Arg2*	ATACAGGGTTGCTCTCAGCATTG	CGTAGCCTTGGCCTCTTCCT
*Asns*	GTGTTCAGAAGCTAAAGGTCTCGTT	GGCGACTTTGCCATTTGG
*Ass1*	TTGGAGAATGCCCGAGTTCTA	GTGTTGCTTCGCGTACTCCAT
*Atf4*	TGGCCATCTCCCAGAAAGTT	GGGAAGAGTTTGCAAGAACGTAA
*Atf5*	TGCCCTCTCCGCAACTTC	CATCTGTTCCAACTCCTTCTTGAG
*Atf6A*	CGTATTCTCCAGGGTGCTCTAGAA	ACTCCCTGAGTTCCTGCTGATACT
*Atf6B*	CCGAAGGGACCACCTGTTG	GCCATGGCAGGCATCAC
*Cdkn1A*	TGGAACTTCGACTTCATCACTGA	CAAGGCCTCGCACACGTT
*Cebpg*	GCTGAACGGGCTAACTGTCACT	GCTGCGGAACCTGCTGTAAG
*Ern1*	CCTGACGAAACTTCCCTTTACG	CATGTAGAGAATTCCATCCGAACTT
*Gadd153*	GAAATGAGGAGGAGTCAAAAACCTT	GCTCTGGGAGGTGTGTGTGA
*Gadd45A*	CAGAAGACTGAAAGGATGGATAAGG	GGTGATTGTGCGCTGACTCA
*Sars*	GATGATGGACAAGGTGGAGTTTG	AGTTCTCCAGGATGGCACAGA
*Sesn2*	AAGACCACCCGAAGAATGTACAA	GCAAGTTCACGTGGACCTTCTC
*Slc3a2*	TCAGCGAGGATAGGCTCTTGA	GGACAGGTACGAGCTAGTTAACAACA
*Slc7a1*	GGTGGTCACGGCCATCAT	GGAAGGGCACCTTGAAGGA
*Slc1a5*	CAAGGAGGTGCTCGATTCGT	TTGAATGGCCTCTCCGAGTAG
*Xbp1*	GAATGGCCCTTTATCATTTCTCTTC	AAGCAAACTAAAGGACCTAAAATCAGA

### Western blotting

Protein was extracted from 100 mg LD muscle by homogenization in extraction buffer containing 150 mM NaCl, 50 mM HEPES, 2.5 mM EDTA, 10% Glycerol, 1% Triton, and protease and phosphatase inhibitor cocktails (Roche, UK). A sample of whole homogenate and supernatant (generated by centrifugation at ~15,000 g for 10 minutes) were transferred to an equal volume of 2x Laemmli loading buffer. Constant protein (50 µg for LD muscle samples, 5 µg for muscle cells) was loaded and separated on a 4–15% precast acrylamide gel (Criterion TGX, BioRad) and wet transferred to nitrocellulose membranes, with ponceau staining used to confirm the equal loading of protein. Whole homogenate samples were probed using anti-ATF4 (Cell Signaling; #11815) and supernatant samples were probed using anti-ASNS (Atlas Antibodies; HPA029318) followed by anti-rabbit secondary (GE Healthcare Life Science). Bands were visualized using ECL detection reagent (GE Healthcare Life Science). Densitometry of band intensity was conducted to determine protein expression levels.

### Porcine myoblast cells for ASNS and ATF4 antibody validation

Porcine myogenic precursor cells were a kind gift from Dr Paul Loughna (University of Nottingham, UK) and were originally isolated from the *Semimembranosus* muscle as previously described^21^. Confluent porcine cells were cultured in Dulbecco’s Modified Eagles Medium (DMEM) with 20% fetal calf serum, 100 units/ml penicillin, 0.1 mg/ml streptomycin and 2.5 µg/ml Amphotericin B. Cells were treated with 250 nM Thapsigargin for 8 hours to induce expression of endoplasmic reticulum stress proteins. All cell culture reagents were purchased from Sigma Aldrich UK. Cells were harvested directly into 1x Laemmli loading buffer including protease and phosphatase inhibitors (Roche) and stored at −20C until use. Samples were used for western blotting as described above.

### Statistical analysis

Statistical analyses on qPCR and Western blotting data were performed using Genstat (13^th^–15^th^ edition). Changes in relative mRNA transcript abundance were assessed for significant treatment (BA, GH or control), time (1, 3, 7, 13 and 27 days) and treatment x time interactions using a two-way analysis of variance (ANOVA). Post-hoc Dunnett’s tests were performed to compare BA and GH group means to the control mean, whereas Bonferroni tests were used for significant time x treatment interactions. Significance was accepted if *p* < 0.05. Relative mRNA expression is presented as mean ± standard error of the mean (SEM).

## Results

### Ractopamine increases expression of tRNA charging and amino acid biosynthesis genes

**Figure 1:**
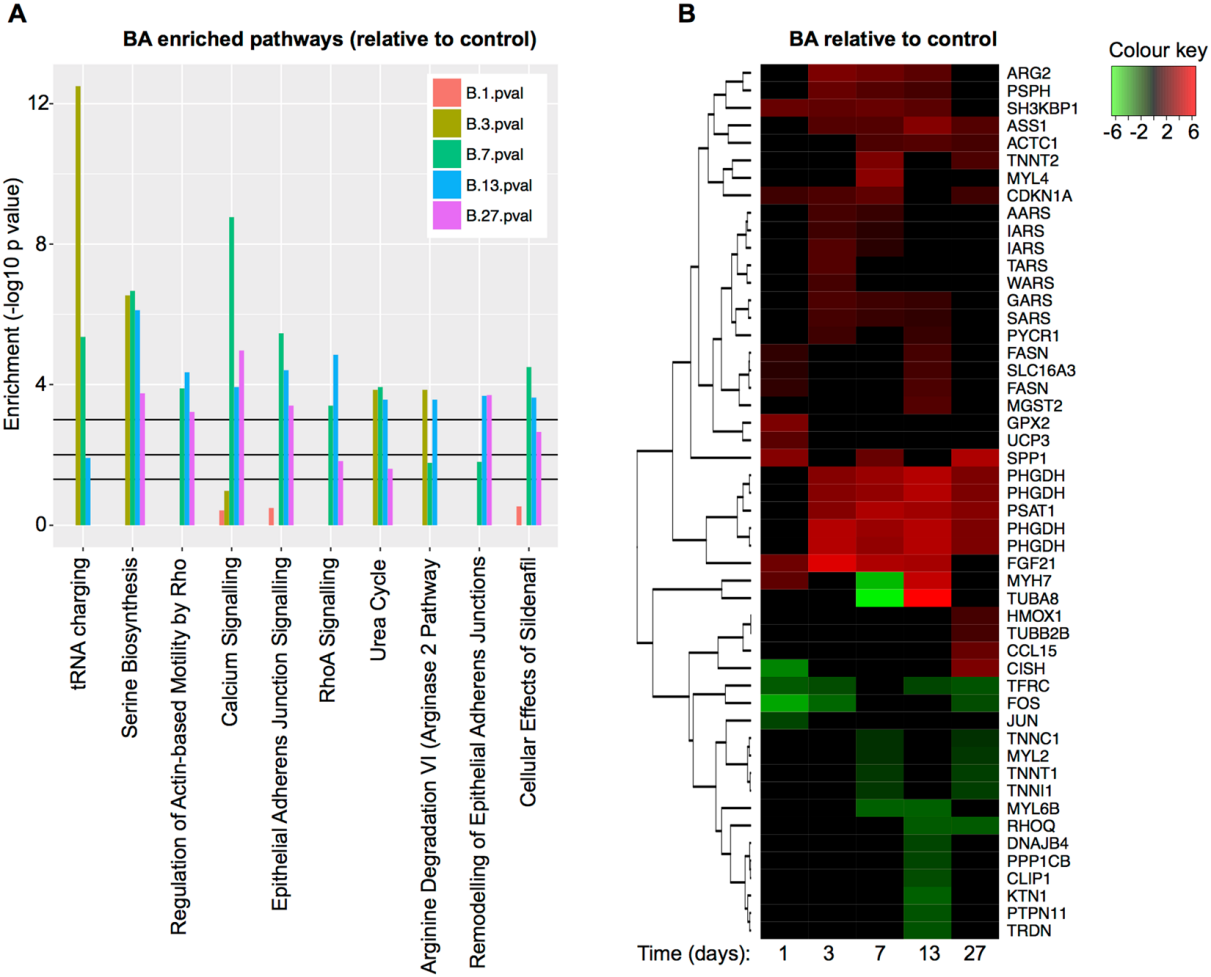
Inferred pathway enrichment analysis for BA treated animals. (**A**) Enriched pathways are ranked from left-to-right by mean –log10 *p* value. The 3 black lines from bottom-to-top depict *p* values of 0.05, 0.01 and 0.001 respectively. Colours of the bar chart are orange (day 1) olive green (day 3), bright green (day 7), blue (day 13) and purple (day 27). (**B**) Heat map to show top changing genes identified by inferred pathway analysis. Red and green respectively indicate treatment-induced increases and decreases in gene expression relative to that of the control animals. The intensity of red and green denote the significance (−log10 *p* value) of the change in expression (see colour key). Each column represents a time point (in days) following the initiation of treatment. *n* = 3 per treatment per time point.

RNA-sequencing analysis revealed that BA treatment had greater effects than GH and inferred pathway enrichment analysis (IPA) revealed that four of the top ten enriched pathways for BA were involved in tRNA charging, serine biosynthesis or arginine metabolism (Fig. 1A). Of the top ten enriched pathways for BA, none displayed significant enrichment prior to day 3. The most significantly enriched pathway for BA treatment was tRNA charging (Fig. 1A), with several aminoacyl-tRNA synthetase genes (*Aars*, *Iars*, *Tars*, *Wars*, *Gars*, *Sars*) being strongly up regulated, particularly at day 3 (see heat map in Fig. 1B). The serine biosynthesis pathway genes (*Phgdh*, *Psat1*, *Psph*) formed the second most significantly enriched pathway, which again were particularly evident from day 3 onwards (Fig. 1A,B) and agreed with our previous microarray analyses^14^ where we saw increases in *Phgdh* and *Pck2* mRNA and protein in these same animals. Enriched genes in the Calcium Signalling pathway mainly related to structural and contractile proteins in muscle (e.g. *Actc1* and *Myl4* – Fig. 1B and Supplementary Table 1), with many of those down-regulated by BA being associated with slow muscle fibres (e.g. *Myh7*, *Tnnt1*, *Tnnc1* and *Tnnt2*). Genes involved in the urea cycle (*Arg2* and *Ass1*) and arginine degradation (*Arg2* and *Pycr1*) also displayed significant enrichment from day 3 onwards. These analyses therefore indicated that day 3 was a time point whereby metabolic adaptations to the muscle-modifying drug, Ractopamine, were most pertinent.

### Ractopamine increases skeletal muscle expression of Asparagine Synthetase (ASNS)

**Figure 2:**
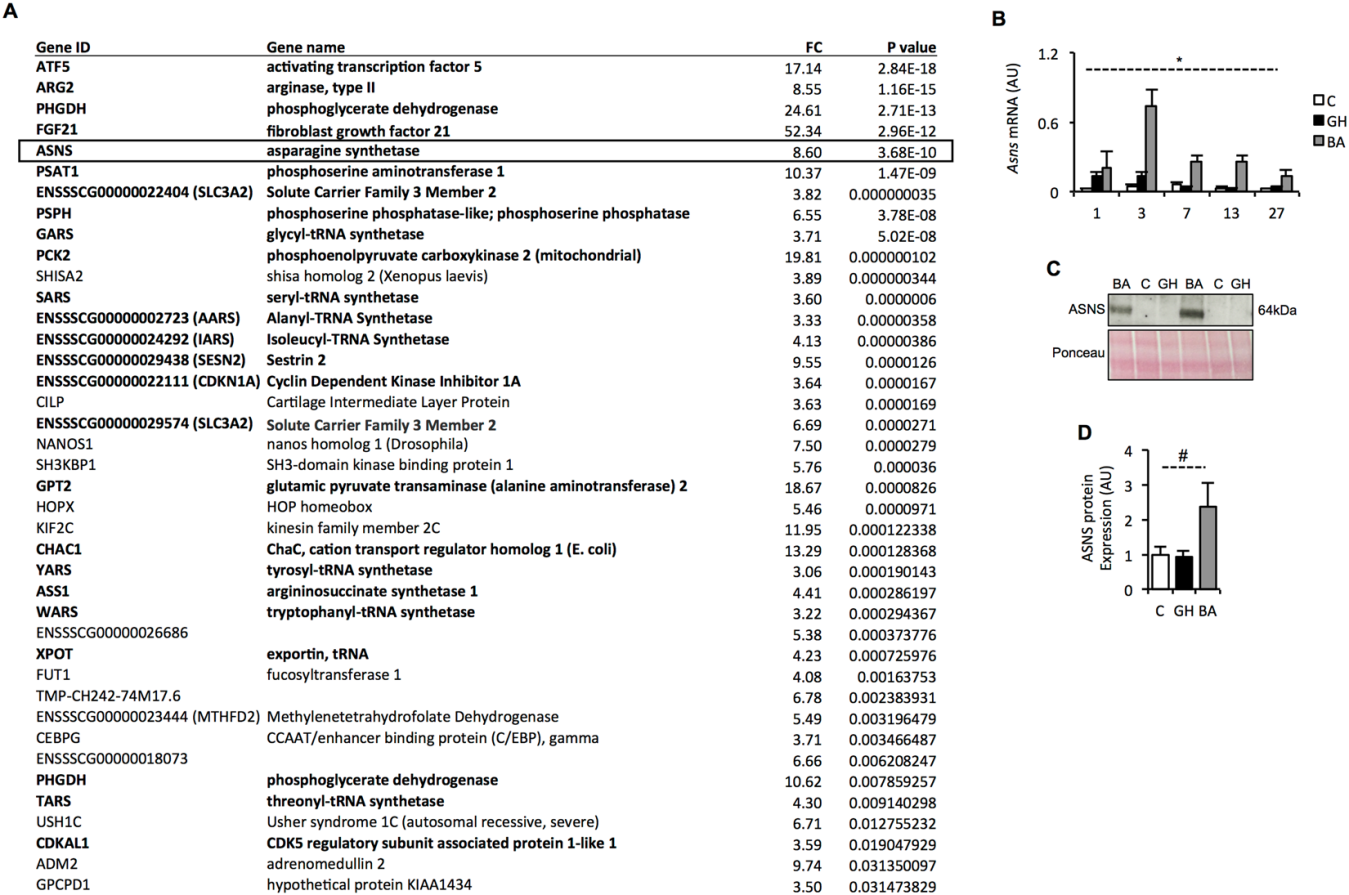
Identification of Asparagine Synthetase (Asns) as a novel BA responsive gene. (**A**) Genes displaying >3-fold increase in expression with BA treatment at day 3 (relative to control) measured by RNA-sequencing. Genes ranked by significance (*p* < 0.05). (**B**) qPCR validation of *Asns* expression in all samples (Control, BA and GH; total *n* = 164). *p < 0.001 for time x treatment interaction (see Supplementary Table 2 for *post hoc* Bonferroni test comparisons). (**C**) Representative image of western blot for ASNS in control, BA and GH treated LD muscles at day 7 (see supplementary Figure 1 for full blot and gel). (**D**) Quantitation of ASNS protein abundance at day 7 from western blot. ^#^p < 0.05 (ANOVA).

IPA analysis (Fig. 1), as well as our previous microarray analyses^14^, directed our interest to genes increasing in expression at day 3 of BA treatment. A list of genes that showed a greater than 3-fold increase in expression by BA treatment at day 3 (p < 0.05) are displayed in Fig. 2A and affirm the results of the IPA analysis. The 24 genes highlighted in bold (Fig. 2A) are largely involved in amino acid biosynthesis and transport as well as protein translation. Ingenuity Knowledge Base analysis, together with searching the published literature, indicated that these genes are highly indicative of an integrated stress response (ISR)^22^. Further indication of an ISR is that the most significantly up regulated gene at day 3 was activating transcription factor 5 (*Atf5*), which binds to promoters of integrated stress responsive genes^23^.

The increased *Asns* transcript abundance (identified by RNA-sequencing analysis) at day three was particularly noteworthy given that this enzyme typically displays very low basal expression in most tissues^24^. Confirmation of *Asns* mRNA expression by qPCR revealed a potent increase in mRNA abundance in response to BA treatment, peaking at day 3 but remaining elevated above control levels throughout the entirety of the 27-day study period (treatment x time interaction: p < 0.001; Fig. 2B). Furthermore, western blot analysis of ASNS protein expression at day 7 (the time point following the peak in mRNA abundance) showed induction of ASNS protein in skeletal muscle of pigs treated with Ractopamine (Fig. 2C,D), although the magnitude of response was quite variable between individual pigs as is reflected in the larger error bar (SEM). Unlike BA treatment, GH did not increase ASNS protein expression on day 7 (Fig. 2C), but it did induce a transient increase in *Asns* mRNA expression on days 1 and 3 (Fig. 2B), with expression reducing to control levels thereafter.

### Ractopamine perturbs metabolic homeostasis

**Figure 3:**
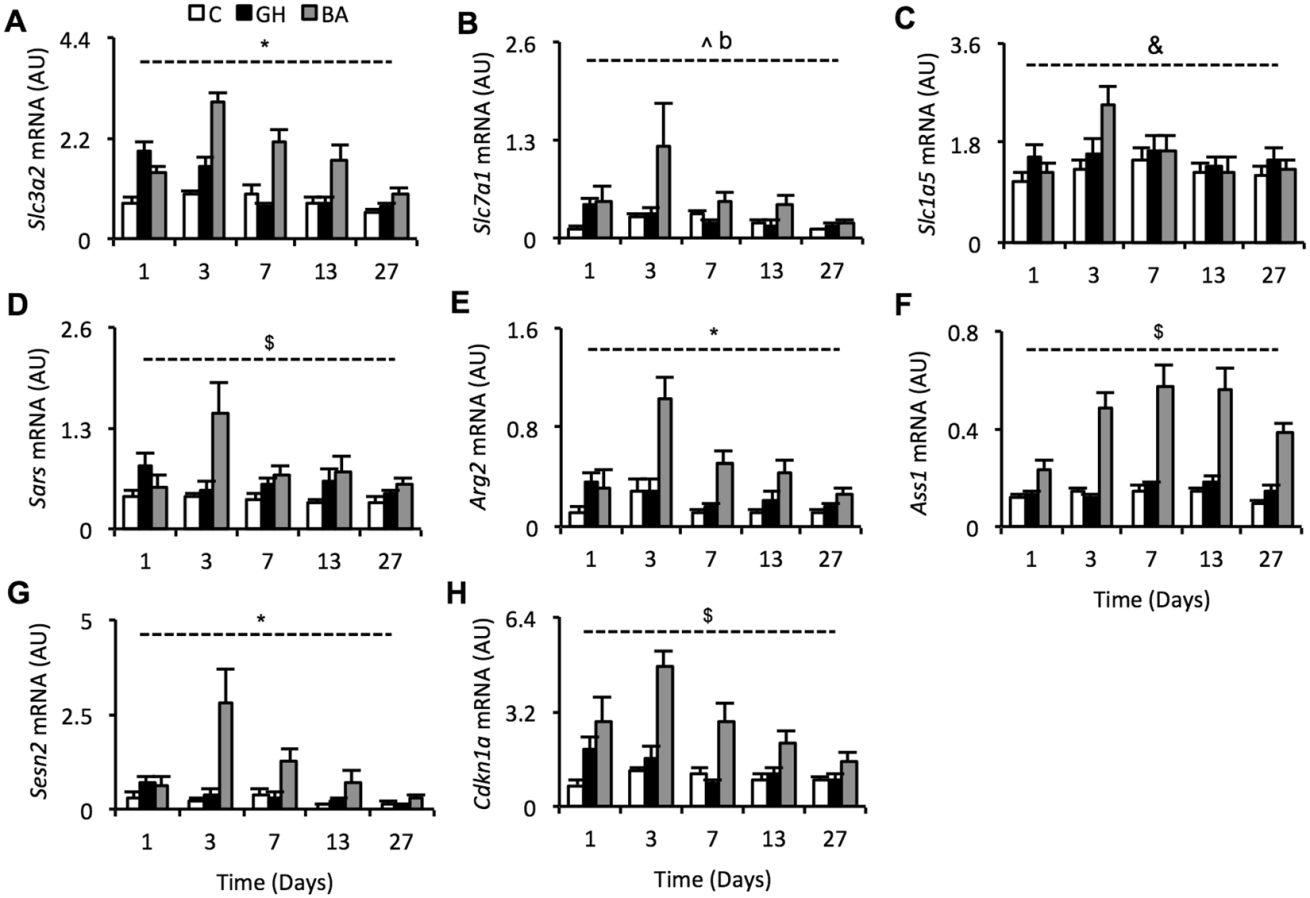
Validation of amino acid transport, amino acid biosynthesis and tRNA charging gene expression. Messenger RNA expression of the following genes were validated by qPCR on all 164 samples (**A**) solute carrier family 3 member 2, (**B**) solute carrier family 7 member 1, (**C**) solute carrier family 1 member 5, (**D**) seryl-tRNA synthetase, (**E**) arginase 2, (**F**), argininosuccinate synthetase 1, (**G**) sestrin-2 and (**H**) cyclin-dependent kinase inhibitor 1A. *Indicates a treatment-time interaction with *p* < 0.001, ^$^indicates a treatment-time interaction with *p* < 0.005, ^indicates a treatment effect with *p* < 0.01, ^&^indicates a treatment effect with *p* < 0.05, b indicates a significant Dunnetts test with *p* < 0.01 for BA vs. control (GH vs. control  = NS). For genes with significant interactions, see Supplementary Table 2 for *post hoc* Bonferroni test comparisons. Data are mean ± SEM. Days 1, 3, 7 and 13 have *n* = 10 whilst day 27 has *n* = 15. White bars = control, black bars = GH (growth hormone), grey bars = BA (beta-adrenergic agonist).

Expression levels of a subset of genes displayed in Fig. 2A were validated by qPCR (Fig. 3A–H) to confirm the widespread increase in expression of genes involved in an ISR and metabolic homeostasis. Messenger RNA abundance of solute carrier genes (*Slc3a2*, *Slc7a1*, *Slc1a5*), which are involved in amino acid transport, were increased by BA treatment (Fig. 3A–C; see figure legend for stats). The day 3 increase in expression of seven tRNA synthetases was affirmed by measuring *Sars* mRNA abundance (Fig. 3D), which displayed an obvious increase in expression specifically at day 3 with BA treatment (treatment x time interaction: p < 0.005). Arginase 2 (*Arg2*) and argininosuccinate synthase 1 (*Ass1*), which are involved in arginine metabolism, also displayed increased mRNA expression with BA treatment (treatment x time interaction of p < 0.001 and p < 0.005, respectively; Fig. 3E,F). The stress responsive genes, sestrin-2 (*Sesn2*) and cyclin-dependant kinase 1 alpha (*Cdkn1a*), which are both negative regulators of cellular growth, were also strongly increased by BA treatment (treatment x time interaction of p < 0.001 and p < 0.005, respectively; Fig. 3G,H). As observed for *Asns* mRNA expression (Fig. 2B), GH induced a transient increase in mRNA, mainly on day 1, for the majority of these genes (all except *Ass1*), with expression reducing to control levels thereafter (Fig. 3A–H).

### Ractopamine does not alter expression of Atf4 but it does increase Atf5 mRNA

**Figure 4:**
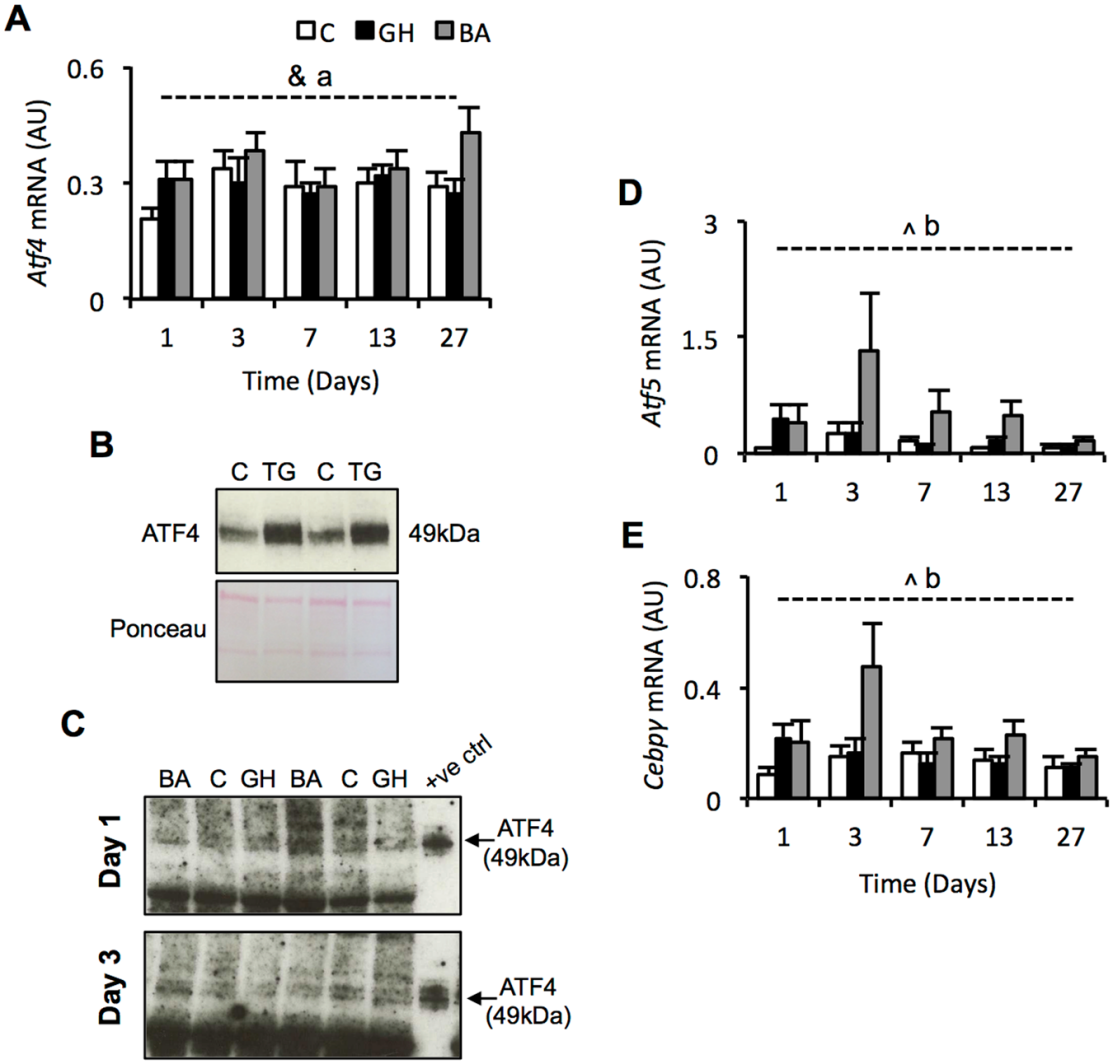
Expression of ATF4 is not altered by BA treatment but Atf5 mRNA is increased. (**A**) mRNA abundance of activating transcription factor 4. (**B**) Validation of anti-ATF4 antibody to detect ATF4 from protein extracted from cultured porcine muscle cells treated with or without thapsigargin (TG) - see supplementary figure 2 for full blot and gel. (**C**) Representative western blots attempting to detect ATF4 protein in muscle samples from BA, control or GH treated pigs (see supplementary Figure 3 for full blots). (**D**) qPCR validation of activating transcription factor 5 gene expression. (**E**) CCAAT/enhancer binding protein gamma mRNA abundance measured by qPCR. ^indicates a treatment effect with *p* < 0.01, ^&^indicates a treatment effect with *p* < 0.05, ^a^indicates a significant Dunnetts test with *p* < 0.05 for BA vs. control (GH vs. control = NS), ^b^indicates a significant Dunnetts test with *p* < 0.01 for BA vs. control (GH vs. control = NS). Data is mean ± SEM. Days 1, 3, 7 and 13 have *n* = 10 whilst day 27 has *n* = 15. White bars = control, black bars = GH (growth hormone), grey bars = BA (beta-adrenergic agonist).

The BA responsive genes identified herein are all highly suggestive of a perturbation to metabolic homeostasis, particularly amino acid metabolism and protein translation. Ingenuity Knowledge Base analysis, together with searching the published literature, indicated that more than 20 of the genes increased by BA treatment at day 3 are documented targets of activating transcription factor 4 (*Atf4*), a central component of the integrated stress response (ISR)^22^. Messenger RNA expression of *Atf4* showed little effect of treatment (Fig. 4A), which is perhaps unsurprising because ATF4 protein expression is largely regulated post-transcriptionally. Due to the well-known difficulties associated with measuring ATF4 protein expression *in vivo*, we first generated an ATF4 positive control by treating primary porcine myoblasts with an ATF4-inducing compound, thapsigargin. Using protein from these cells enabled us to confirm that the antibody used herein was capable of detecting porcine ATF4 in skeletal muscle cells, as shown in Fig. 4B. Next, we probed pig muscle samples from days 1 and 3 with this ATF4 antibody. These time points were chosen because they preceded and coincided, respectively, with the peak in mRNA expression of the suspected ATF4 target genes. We were unable to confidently detect a quantifiable band representing ATF4 (Fig. 4C), most likely due to a very low abundance of the protein. We conclude that, using the methods employed herein, ATF4 protein expression was probably not changed by BA treatment.

Interestingly, RNA-sequencing analysis identified activating transcription factor 5 (*Atf5*) as the most significantly increased gene on day 3 (Fig. 2A). This was confirmed by qPCR, which revealed an expression profile similar to genes in Fig. 3, with the increased expression peaking at day 3 (treatment effect: p < 0.01). However, commercially available antibodies against ATF5 were unable to detect porcine ATF5 (data not included). Expression of CCAAT/enhancer binding protein gamma (*Cebpg*) mRNA, a known co-factor of activating transcription factors, was also increased by BA treatment (treatment effect: p < 0.05); whereas expression of *Atf6* and other known endoplasmic reticulum stress-response genes, including *Gadd45a*, *Gadd153*, *Xbp1*, and *Ern1*, were not significantly altered by BA treatment (p > 0.05; Supplementary Fig. 4A–F).

## Discussion

In line with NC3R guidelines to reduce the use of animals^15^, we used samples generated by a previously published animal trial^14^ to conduct RNA sequencing and inferred pathway enrichment analysis (IPA), enabling us to obtain better coverage of the skeletal muscle transcriptome in pigs treated with BA or GH. In doing so, we showed that BA treatment had greater effects than GH and identified novel biological events associated with BA treatment. Identifying mechanisms underpinning the actions of anabolic agents, such as BA, is crucial for the development of novel muscle anabolic agents for use in livestock production and for the treatment of muscle wasting diseases.

### Asparagine synthetase (ASNS) induction by BA treatment

We identified the induction of the asparagine synthetase (ASNS) protein in skeletal muscle of pigs administered with a BA, which was not identified in the previous report using these samples^14^. With the exception of the pancreas, basal expression of *Asns* is typically very low in all tissues^24^, as was evident in skeletal muscle herein. Expression of *Asns* is often associated with amino acid limitation^24^. ASNS converts aspartate and glutamine to asparagine and glutamate^24^, with the majority of newly synthesized asparagine being utilized for protein synthesis^25^. In cultured cells, knockdown of *Asns* expression or deprivation of asparagine causes impaired growth^26^. Whether ASNS expression *per se* can influence the protein synthetic capacity of skeletal muscle warrants further investigation, but falls outside the scope of this investigation. The increased ASNS protein expression might contribute to the increased skeletal muscle protein synthesis that is often reported during treatment with BA^1,4^. Alternatively, the BA-mediated induction of ASNS might occur in response to amino acid limitation within the skeletal muscle, perhaps caused by an increased reliance on asparagine or other conditionally essential amino acids. Fast-growing poultry are often fed diets high in conditionally essential amino acids because endogenous synthesis of these amino acids is thought to become rate limiting during rapid growth^27^. It would be interesting to examine whether supplementation with conditionally essential amino acids would ablate the BA-mediated induction of ASNS expression and whether such a strategy would improve the growth response of pigs fed a BA. The implication of enhancing BA-mediated growth in pigs would be substantial in the effort to avoid a global food security crisis by 2050^3,28^.

Altered expression of ASNS was not identified by previous BA transcriptomic studies in rodents^9,10^. Differences in inter-species metabolic stability^29,30^ may cause transcriptomic responses controlling amino acid metabolism to differ between large mammals and small rodents^9,10^.

### Activating transcription factors (ATF4 and ATF5)

Perhaps the most well studied mechanism controlling *Asns* gene expression is the composite CCAAT/enhancer-binding protein (CEBP)-activating transcription factor (ATF) response element (CARE) located in the *Asns* promoter, which is aptly bound by ATF-CEBP heterodimers. The RNA-sequencing analysis identified *Atf5* as the most significantly up regulated gene at day 3 of BA treatment (which was not reported in our previous publication using these samples^14^), drawing our attention to this as a potential mechanism for the increased *Asns* expression. Over-expression of ATF4 or ATF5 (which have high sequence similarity^31^) has been shown to increase *Asns* promoter activity^31–34^. Protein expression of ATF4 and ATF5 is post-transcriptionally controlled by the phosphorylated form of elongation initiation factor 2 (p-eIF2)^24^. The BA-mediated increase in *Atf5* mRNA expression was confirmed by qPCR but commercially available antibodies could not detect porcine ATF5 protein (data not shown). There was little effect of BA treatment on *Atf4* mRNA expression, and whilst we could measure ATF4 protein in cultured porcine myoblasts, we were unable to confidently detect ATF4 protein in pig skeletal muscle. The short half-life of ATF4 (~30 minutes)^35^, and the non-pathological setting in which we were examining its expression, likely made ATF4 protein abundance difficult to determine. ATF4 has recently been shown to be regulated by phosphorylation^36^, but we were unable to measure phospho-ATF4, so cannot rule out that BA-induced effects could potentially be via phosphorylation of ATF4. Whilst we were unable to measure changes in ATF4 protein expression, the sheer number of known ATF4 target genes (*Asns*^34,37^, *Phgdh*^37,38^, *Psat1*^38^, *Psph*^38^, *Pck2*^39,40^, *Gpt2*^40^, *Wars*^37^, *Sars*^37^, *Gars*^37^, *Tars*^41^, *Slc7a1*^37,41^, *Slc3a2*^37^, *Fgf21*^42,43^, *Sesn2*^40,44^, *Cdkn1a*^45^, *Chac1*^40^, *Atf5*^40^ and *Cebpg*^40^) that displayed a >3-fold increase in expression by BA treatment at day 3 is highly suggestive of ATF involvement in this BA-mediated integrated stress response.

### Ractopamine increases skeletal muscle expression of aminoacyl-tRNA synthetase genes

The most significantly enriched pathway during BA treatment was tRNA charging. A strong increase in expression of six aminoacyl-tRNA synthetases (*Aars*, *Gars*, *Iars*, *Sars, Tars*, *Wars, Yars*) on day 3, accounted for this strong pathway enrichment. Aminoacyl-tRNA synthetases (ARS) catalyse the attachment of specific amino acids to their respective tRNA, thus regulating both the protein synthetic capacity of the cell and the accuracy of protein assembly^46^. Elevated expression of ARS has been documented previously in rapidly proliferating cell types that exhibit augmented rates of protein synthesis, such as cancer cells^46,47^. It remains unknown, however, whether increased expression of ARS drives protein synthesis or whether ARS expression is dictated by the protein synthetic demand of the cell^46^. BA are well known to stimulate changes in skeletal muscle protein metabolism^4^, but a role for differential ARS expression during increased myofibrillar protein accretion has yet to be investigated. Previous transcriptomics studies failed to identify this co-ordinated increase in expression of multiple ARS genes by BA treatment^9,10^, possibly because these changes peaked at a time point (day 3) not used in any of these previous studies^9,10^.

### BA mediated increased mRNA expression of Sestrin-2 and Cdkn1a

Sestrin-2 (*Sesn2*) and cyclin-dependent kinase inhibitor 1A (*Cdkn1a*) mRNA expression were both increased by BA treatment. SESN2 is an inhibitor of mTOR-mediated cell growth^44,48,49^, while CDKN1A is an inhibitor of the cyclin dependant kinases that drive cell proliferation^50^. Transcriptomic studies in rodents have previously shown increased *Cdkn1a* mRNA expression with BA treatment, but these studies did not identify changes in *Sesn2* expression^9,10^. The sustained increase in transcript abundance of *Sesn2* and *Cdkn1a* is highly indicative of cellular stress, inhibition of growth and cell survival^45,48,49^. This is somewhat counterintuitive following administration of a widely reported “anabolic” agent and therefore might present a previously unrecognised negative feedback mechanism to prevent unsustainable growth. *Sesn2* and *Cdkn1a* genes are also transcriptionally induced during amino acid deprivation^40,45^.

## Conclusion

The BA-mediated increase in expression of ASNS (mRNA and protein) and a concomitant increase in mRNA expression of several tRNA-synthetases, *Sesn2* and *Cdkn1a*, is highly indicative of a perturbation to metabolic homeostasis (i.e. an integrated stress response), particularly relating to amino acid biosynthesis and protein translational capacity. On-going work is examining the role of these genes in the regulation of muscle mass. Hence, this re-analysis of the muscle transcriptomic response to anabolic agents (BA and GH) using an improved methodology (RNA sequencing) has enhanced the transcriptome coverage and identified a number of BA-responsive genes not previously identified using microarray, suggesting that an integrated stress response is a potential novel mechanism for BA-induced muscle growth.

## Electronic supplementary material

Supplementary tables and figures
