# Author Correction: The Beta-adrenergic agonist, Ractopamine, increases skeletal muscle expression of Asparagine Synthetase as part of an integrated stress response gene program

**DOI:** 10.1038/s41598-019-43807-1

**Published:** 2019-10-28

**Authors:** David Brown, Kevin Ryan, Zoe Daniel, Molebeledi Mareko, Richard Talbot, Joanna Moreton, Tom C. B. Giles, Richard Emes, Charlie Hodgman, Tim Parr, John M. Brameld

**Affiliations:** 1 School of Biosciences, University of Nottingham, Sutton Bonington Campus, Loughborough, LE12 5RD UK; 2 School of Veterinary Medicine and Science, University of Nottingham, Sutton Bonington Campus, Loughborough, LE12 5RD UK; 3 Advanced Data Analysis Centre, University of Nottingham, Sutton Bonington Campus, Loughborough, LE12 5RD UK; 4 The Roslin Institute and Royal (Dick) School of Veterinary Studies, University of Edinburgh, Midlothian, EH25 9RG UK; 5 Present Address: RenaSci Limited, BioCity, Pennyfoot Street, Nottingham, NG1 1GF UK; 6 Present Address: Genetics, Guy’s Hospital, London, SE1 9RT UK; 7 Present Address: Botswana College of Agriculture, Gaborone, Botswana

Correction to: *Scientific Reports* 10.1038/s41598-018-34315-9, published online 29 October 2018

In the Supplementary Information file originally published with this Article, Table 2 was omitted. This error has been corrected in the Supplementary Information that now accompanies the Article.

