# Supplementary material for: The Beta-adrenergic agonist, Ractopamine, increases skeletal muscle expression of Asparagine Synthetase as part of an integrated stress response gene program
Source: Sci Rep. 2018 Oct 29;8:15915. doi: 10.1038/s41598-018-34315-9 (PMC6206132; doi:10.1038/s41598-018-34315-9)
Supplement: Supplementary file 1 — Supplementary tables and figures [file 41598_2018_34315_MOESM1_ESM.pdf]

Supplementary Figures for "The Beta-adrenergic agonist, Ractopamine, increases skeletal muscle expression of Asparagine Synthetase as part of an integrated stress response gene program"

David M. Brown[1,5], Kevin J.P. Ryan[1,6], Zoe C.T.R. Daniel[1], Molebeledi H.D. Mareko[1,7], Richard Talbot[4], Joanna Moreton[3], Tom C. Giles[3], Richard D. Emes[2,3], T. Charlie Hodgman[1], Tim Parr[1], John M. Brameld[1*]

Supplementary Table 1. Genes within the Calcium signalling pathway altered by Ractopamine treatment

Day	Gene symbol (direction of regulation by Ractopamine) and gene name
Day 1	MYH7 (DOWN) – Myosin Heavy Chain Slow Isoform
Day 3	ATF4 (UP) – Activating Transcription Factor 4/ cAMP-Response Element-Binding Protein 2<br>TPM1 (UP) - Tropomyosin 1
Day 7	TNNT1(DOWN) - Troponin T1, Slow Skeletal Type<br>TNNC1 (DOWN) - Troponin C1, Slow Skeletal And Cardiac Type<br>TNNT2 (UP) - Troponin T2, Cardiac Type<br>MYL4 (UP) - Myosin Light Chain 4, Embryonic Muscle/Atrial Isoform<br>MYH7 (DOWN) – MyHC type I<br>TNNI1 (DOWN) - Troponin I1, Slow Skeletal Type<br>ACTC1 (UP) - Actin, Alpha, Cardiac Muscle 1
Day 13	TRDN (DOWN) – Triadin or CPVT5<br>MYH7 (UP) - – MyHC type I<br>ACTC1 (UP) - Actin, Alpha, Cardiac Muscle 1<br>PNCK (UP) - CaM Kinase I Beta<br>ACTA1 (UP) - Actin, Alpha 1, Skeletal Muscle
Day 27	TNNT1 (DOWN) - Troponin T1, Slow Skeletal Type<br>TNNC1 (DOWN) - Troponin C1, Slow Skeletal And Cardiac Type<br>TNNT2 (DOWN) - Troponin T2, Cardiac Type<br>TNNI1 (DOWN) - Troponin I1, Slow Skeletal Type<br>ACTC1 (UP) - Actin, Alpha, Cardiac Muscle 1

Supplementary Table 2. Post hoc Bonferroni test comparisons for QPCR data (genes with significant time x treatment interactions)

**Time (day)**	**Treatment**	**Asns**	**Slc3a2**	**Sars**	**Arg2**	**Ass1**	**Sesn2**	**Cdkn1a**
**1**	**Cont**	0.0269 a	0.756 ab	0.4155 a	0.1204 ab	0.1176 a	0.3119 a	0.735 a
**1**	**GH**	0.1300 a	**1.885 df**	0.8308 ab	0.3641 ab	0.1314 a	0.7087 a	1.959 ab
**1**	**BA**	0.1989 a	1.440 bcdef	0.5510 a	0.3033 ab	0.2364 ab	0.6125 a	2.830 b
**3**	**Cont**	0.0499 a	0.961 abc	0.4277 a	0.2769 ab	0.1425 a	0.1968 a	1.214 ab
**3**	**GH**	0.1281 a	1.556 bcdef	0.5038 a	0.2949 ab	0.1147 a	0.3909 a	1.613 ab
**3**	**BA**	**0.7353 b**	**3.017 g**	**1.4949 b**	**1.0396 c**	**0.4868 c**	**2.8058 b**	**4.748 c**
**7**	**Cont**	0.0596 a	0.995 abcd	0.3651 a	0.1212 ab	0.1461 a	0.3343 a	1.075 ab
**7**	**GH**	0.0328 a	0.701 ab	0.5671 a	0.1620 ab	0.1696 a	0.3021 a	0.757 a
**7**	**BA**	0.2618 a	**2.087 f**	0.6842 a	0.5136 b	**0.5699 c**	1.2558 a	2.831 b
**13**	**Cont**	0.0338 a	0.777 ab	0.3477 a	0.1235 ab	0.1426 a	0.1040 a	0.884 a
**13**	**GH**	0.0317 a	0.767 ab	0.6141 a	0.2220 ab	0.1807 a	0.2175 a	1.072 ab
**13**	**BA**	0.2555 a	**1.737 cdef**	0.7345 a	0.4265 ab	**0.5654 c**	0.7203 a	2.129 ab
**27**	**Cont**	0.0232 a	0.610 a	0.3533 a	0.1196 a	0.0968 a	0.1686 a	0.882 a
**27**	**GH**	0.0367 a	0.740 ab	0.4569 a	0.1553 ab	0.1414 a	0.1389 a	0.925 a
**27**	**BA**	0.1377 a	1.005 abcde	0.5989 a	0.2525 ab	0.3869 bc	0.3123 a	1.546 ab

Data shows the means only. Days 1, 3, 7 and 13 have n = 10, whilst day 27 has n = 15.

Means for each gene with different letters are significantly different ($p<0.05$, post hoc Bonferroni test).

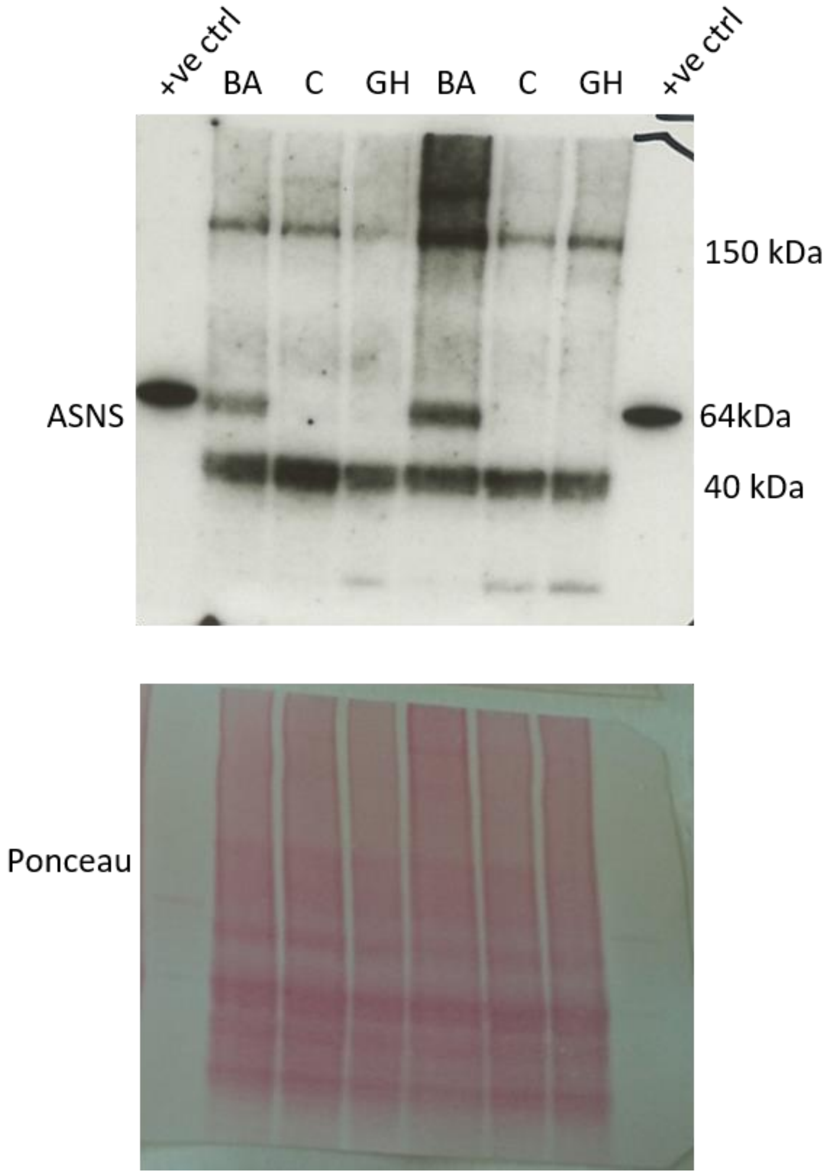

Supplementary Figure 1. Representative images of the full blot and ponceau stained membrane for western blot determination of Asparagine Synthase (ASNS) protein in LD muscle samples from control (C), Beta-Agonist (BA) and Growth Hormone (GH) treated pigs at day 7. The +ve ctrl was untreated porcine myoblast cells.

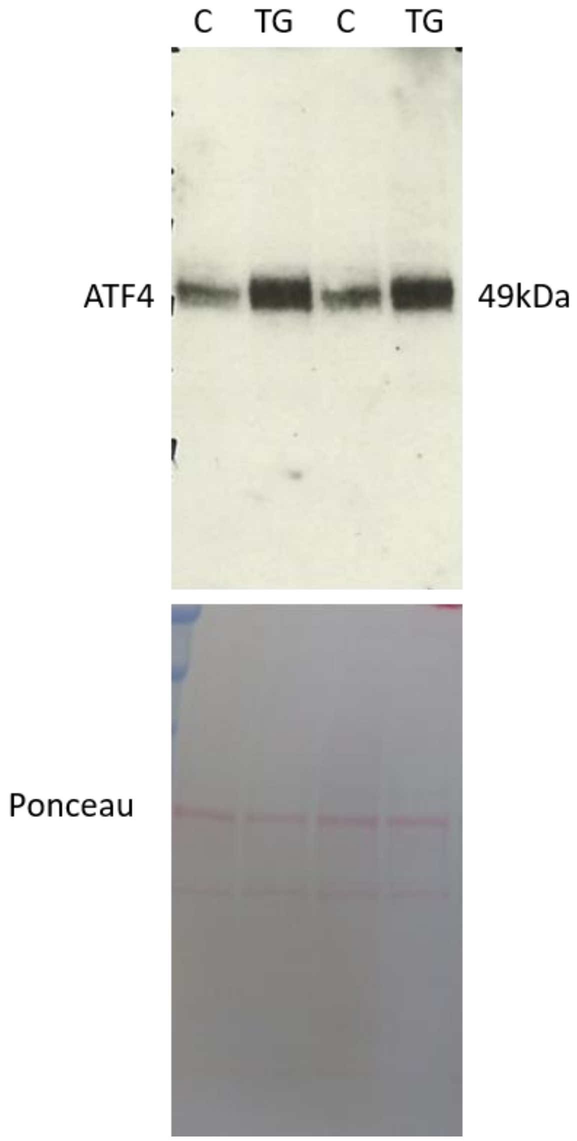

Supplementary Figure 2. Representative images of the full blot and ponceau stained membrane for western blot validation of the anti-ATF4 antibody to detect ATF4 in protein extracted from cultured porcine myoblast cells treated with or without 250nM thapsigargin for 8hrs (C= Control; TG= Thapsigargin treated). A single band was detected in all cells, which was increased in thapsigargin-treated cells.

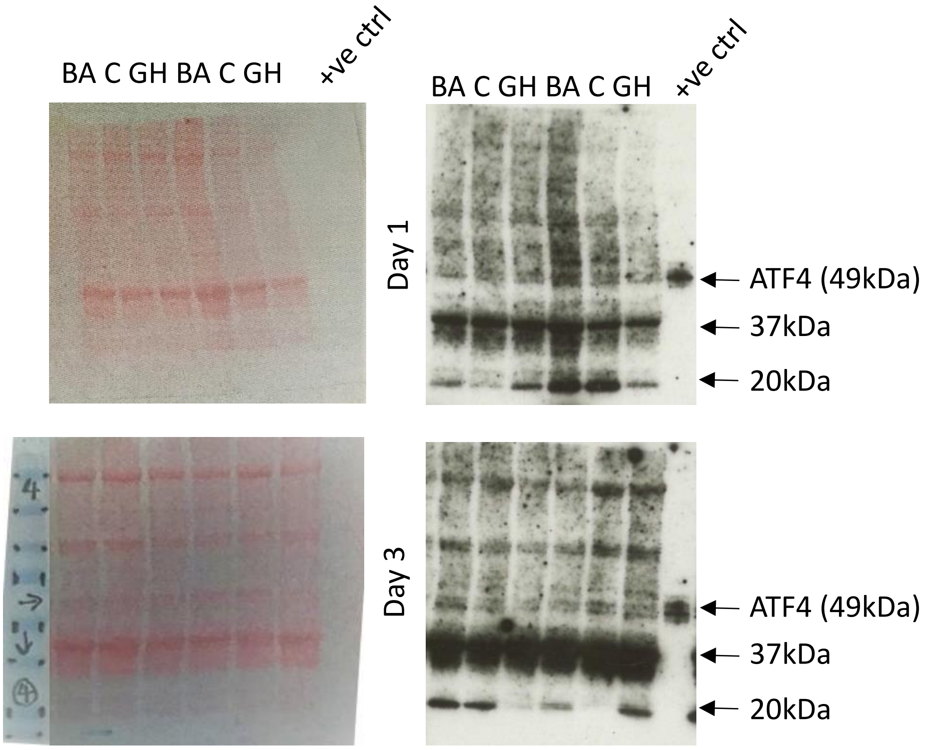

Supplementary Figure 3. Representative images of the ponceau stained membranes (left) and full blots (right) for western blotting to detect ATF4 protein in LD muscle samples from Control (C), Beta-Agonist (BA) or Growth Hormone (GH) treated pigs at days 1 and 3. The +ve ctrl was porcine myoblast cells treated with 250nM thapsigargin for 8hrs where a single clear band is observed. No clear band for ATF4 was detected in any of the pig LD muscle samples.

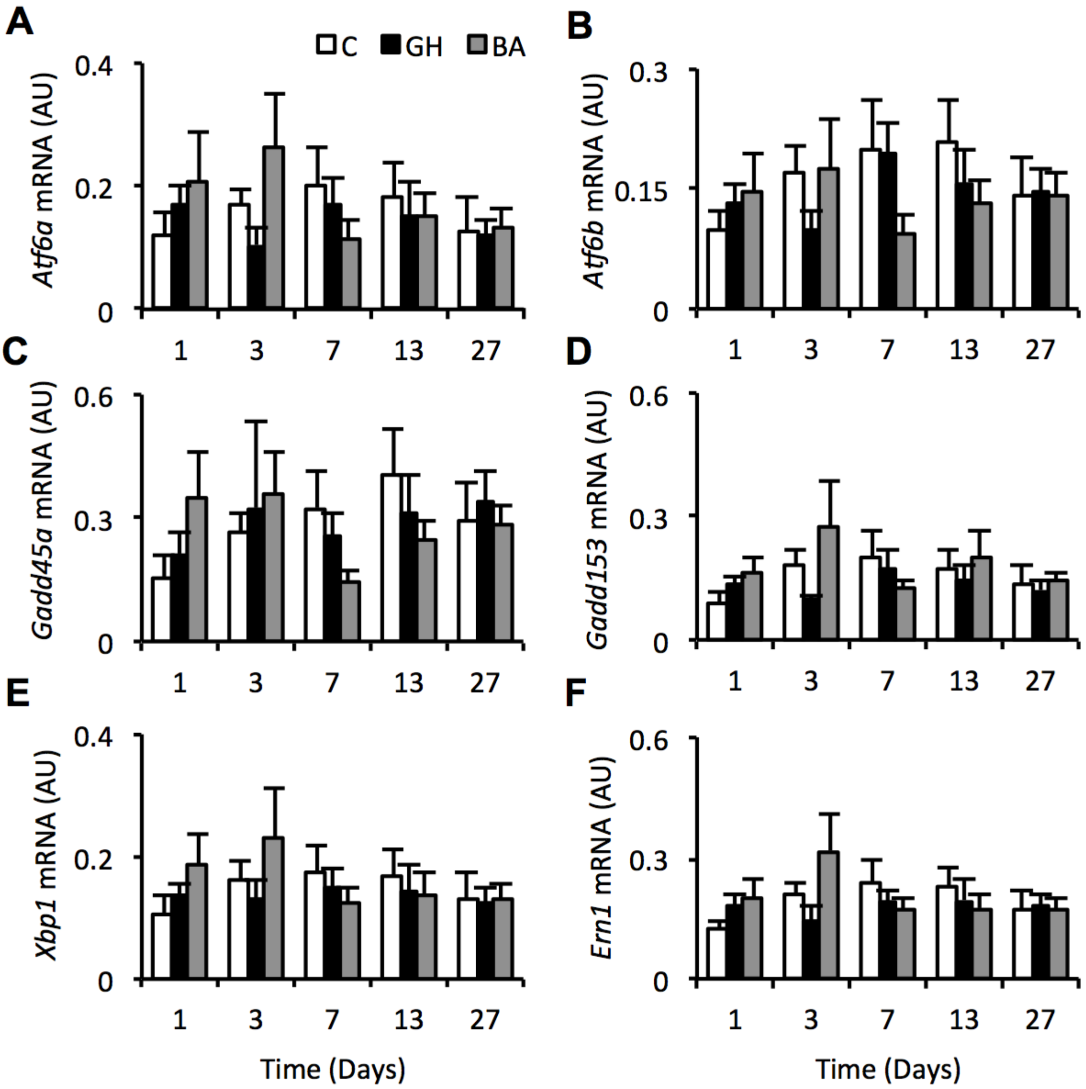

Supplementary Figure 4. Gene expression of endoplasmic reticulum stress responsive genes

Messenger RNA expression of the following genes were validated by qPCR on all 164 samples: (A) activating transcription factor 6a (*Atf6a*), (B) activating transcription factor 6b (*Atf6b*), (C) Growth arrest and DNA damage inducible 45 alpha (*Gadd45a*), (D) Growth arrest and DNA damage inducible 153 (*Gadd153*), (E) X-box binding protein 1 (*Xbp1*), (F) Endoplasmic reticulum to nucleus signalling 1 (*Ern1*).

There were no statistically significant differences in expression levels. Data is mean ± SEM. Days 1, 3, 7 and 13 have n = 10 whilst day 27 has n = 15. White bars = control, black bars = GH (growth hormone), grey bars = BA (beta-adrenergic agonist).
